# Trends in the use of computerized physician order entry by health-system affiliated ambulatory clinics in the United States, 2014–2016

**DOI:** 10.1186/s12913-020-05679-4

**Published:** 2020-09-07

**Authors:** Shira H. Fischer, Robert S. Rudin, Yunfeng Shi, Paul Shekelle, Alejandro Amill-Rosario, Dennis Scanlon, Cheryl L. Damberg

**Affiliations:** 1 RAND Corporation, 20 Park Plaza, Suite 920, Boston, MA 02116 USA; 2 Pennsylvania State University, University Park, State College, PA USA; 3 RAND Corporation, 1776 Main St, Santa Monica, CA 90401 USA; 4 West Los Angeles VA Medical Center, 11301 Wilshire Boulevard, Los Angeles, CA 90073 USA

**Keywords:** CPOE, Computerized provider order entry, Ambulatory clinics, Health systems

## Abstract

**Background:**

Computerized provider order entry (CPOE) can help providers deliver better quality care. We aimed to understand recent trends in use of CPOE by health system-affiliated ambulatory clinics.

**Methods:**

We analyzed longitudinal data (2014–2016) for 19,109 ambulatory clinics that participated in all 3 years of the Healthcare Information and Management Systems Society Analytics survey to assess use of CPOE and identify characteristics of clinics associated with CPOE use.

We calculated descriptive statistics to examine overall trends in use, location of order entry (bedside vs. clinical station), and system-level use CPOE across all clinics. We used linear probability models to explore the association between clinic characteristics (practice size, practice type, and health system type) and two outcomes of interest: CPOE use at any point between 2014 and 2016, and CPOE use beginning in 2015 or 2016.

**Results:**

Between 2014 and 2016, use of CPOE increased more than 9 percentage points from 58 to 67%. Larger clinics and those affiliated with multi-hospital health systems were more likely to have reported use of CPOE. We found no difference in CPOE use by primary care versus specialty care clinics. When used, most clinics reported using CPOE for most or all of their orders. Health systems that used CPOE usually did so for all system-affiliated clinics.

**Conclusions:**

Small practice size or not being part of a multi-hospital system are associated with lower use of CPOE between 2014 and 2016. Less than optimal use in these environments may be harming patient outcomes.

## Background

As hospitals and clinics have moved to adopt electronic health records (EHRs), the use of computerized provider order entry (CPOE) has grown considerably [1]. This upward trend in CPOE use aligns with federal policies designed to encourage the adoption of health information technology (IT) to improve the quality of care; early studies in the hospital setting found that CPOE use was associated with improvements in safety, as well as with improved efficiency and reimbursement [2, 3]. Specifically, the 2009 Health Information Technology for Economic and Clinical Health (HITECH) Act and Meaningful Use offered incentives to eligible providers, including those in ambulatory care settings, to adopt health IT. Since the HITECH Act, there has been a massive increase in EHR adoption in general and CPOE adoption in particular, especially in the hospital setting: the Agency for Healthcare Research and Quality reports that 84% of non-federal acute-care hospitals had implemented an EHR that included CPOE by the end of 2015, which is the most recent published data [4].

In contrast, data are sparse regarding ambulatory practice use of health IT. One form of CPOE is e-prescribing, and at the end of 2015, studies of national samples suggested that slightly more than half of ambulatory practices had adopted EHRs with e-prescribing capabilities [5, 6]. However, little is known about CPOE use beyond medication prescribing as well as the factors associated with adoption in the ambulatory setting.

To understand the current state of CPOE use and recent trends in use by ambulatory practices, we used data from a national survey of health system-affiliated ambulatory clinics to examine current rates of use of CPOE and trends in adoption over a 3-year period and the association between CPOE use and clinic characteristics (practice size, practice type, and participation in a multi-hospital system).

## Methods

CPOE includes computerized ordering of imaging tests, laboratory orders, referrals to other providers, and sometimes also electronic medication prescribing, though e-prescribing is often studied as a separate functionality. In Meaningful Use criteria [7], as well as in the survey data we use (see below), e-prescribing is also measured by separate items. We focus in this paper on the Healthcare Information and Management Systems Society (HIMSS) Analytics Ambulatory Survey (HIMSS Analytics LOGIC™ Market Intelligence Platform) questions that ask about CPOE and not the specific medication ordering questions, and we interpret the survey questions about CPOE to include laboratory and other tests and referrals that a doctor can order.

### Data source

We used three-year panel data (2014–2016) from the annual HIMSS Analytics Ambulatory Survey. This is a companion survey to the original HIMSS hospital survey, which has been used in studies of HIT and CPOE adoption [8–10]. The ambulatory survey includes clinics defined as facilities that provide “preventative, diagnostic, therapeutic, surgical, and/or rehabilitative outpatient care where the duration of treatment is less than 24 hours—and is generally referred to as outpatient care.”

The survey captures information on more than 75% of U.S. health system-associated ambulatory care practices [11]. HIMSS defines a health system as an organization including at least one hospital and its associated nonacute facilities, where “associated” indicates a governance relationship (i.e., owned, leased, or managed by a health system). The survey includes approximately 42,000 clinics across the United States.

### Approach

We limited our datasets to health system-associated ambulatory clinics that deliver primary or specialty care, eliminating imaging centers or other locations where ordering was not expected. We removed observations that were missing entries for predictor variables. When adoption patterns seemed illogical or inaccurate—for example, if a clinic was noted as using CPOE in 2014 and 2016 but not in 2015, or if an answer did not match the number of answer choices, we considered the responses as probably incorrect and dropped the clinic from our sample. This logic-checking resulted in eliminating 2169 records, or about 10%, with a final sample size of 19,109 clinics.

### Clinic characteristics

Using HIMSS survey data, we classified ambulatory clinics according to size (based on number of physicians, dichotomized into 3 or fewer vs. > 3); clinic type (primary versus specialty), and health system type (single hospital or multi-hospital health system).

### Study outcome

We measured two outcomes, CPOE use and the frequency of that use, that were collected in the HIMSS survey. The survey first asks clinics about how EHR software is being used, with a checkbox option for “Clinician Order Entry,” among other options. Within the Clinician Order Entry question response options, respondents could indicate location where CPOE was used (i.e., whether the functionality was available at a clinician station or at the point of care) with both options potentially being selected. We created a single variable that represented overall use, whether the overall question or any location was selected, and we used this variable for most of our analyses.

The second relevant question focused on percent of CPOE use, titled “CPOE - % of Medical Orders Entered by Physicians.” If this option was checked off in the main column, the respondent was asked to estimate a percentage of medical orders that were entered electronically by physicians. There were five options: 1–25% of orders, 26–50% of orders, 51–75% of orders, 76–94% of orders, or > 94% of orders. We used these findings for analyses about percentage of orders.

The survey did not clarify whether CPOE was to include e-prescribing, and there were other medication management questions on the survey, so we did an additional analysis on the questions on electronic medication prescribing (namely, two checkboxes for “E-Prescribing new medications” and “E- Prescribing refill medication requests”) as a comparison, described below.

### Analysis

We computed descriptive statistics, reporting the mean CPOE use rate by year (2014–2016) and by clinic type.

We used a multivariable linear probability model to examine the association between use of CPOE by 2016 and three clinic characteristics available in the HIMSS database: size (based on number of physicians, dichotomized into 3 or fewer vs. > 3); clinic type (primary versus specialty), and whether the clinic was part of a health system, and if so what system type (i.e., single hospital or multi-hospital health system).

As a sensitivity analysis, we examined the factors associated with changes in adoption status. With the subsample of clinics that did not have CPOE in 2014, we used multivariable linear probability models to analyze the relationship between size, clinic type, and health system type and new adoption (i.e., clinics newly adopting CPOE in 2015 or 2016). In all regression analyses, we clustered standard errors at the health system level to account for multiple clinics associated with the same health system.

We then examined use of CPOE by clinics within health systems by calculating the number of health systems with full adoption (all affiliated clinics used CPOE), partial adoption (some clinics used CPOE while others did not), or no adoption (no clinics within a system used CPOE).

As additional analyses, we explored factors predicting use of electronic prescribing of medications, to see if there were different predictors for this related functionality, which is a form of CPOE restricted to medications, and we also compared data exchange over time and among those with CPOE and without.

## Results

In 2016, our study sample had 19,109 clinics within 1548 systems (Table 1). The majority of ambulatory clinics had EHRs (88% by 2014; 96% in 2016). The number of clinics per health system also increased over this time period. The total number of health systems decreased by 109 (almost 7%), with the number of clinics per system increasing, likely reflecting consolidation over the study period. The portion of primary care clinics was stable at around 65%. An increasing proportion of clinics were part of a multi-hospital system, again possibly reflecting mergers and acquisitions. Characteristics of ambulatory clinics and the health systems in which they exist are shown in Table 1.

**Table 1:** Characteristics of Ambulatory Clinics Included in the CPOE Study Sample (2014–2016)

Year	2014	2015	2016
Number of clinics	19,109	19,109	19,109
Number of health systems	1657	1603	1548
Number of clinics per health system (standard deviation)	11.53 (26)	11.92 (27)	12.34 (29)
Number of clinics that are Primary Care (% of total clinics)	12,439 (65%)	12,336 (65%)	12,296 (64%)
Number of clinics in multi-hospital systems (% of total clinics)	13,301 (69%)	13,718 (72%)	13,887 (72%)
Number of physicians per clinic (standard deviation)	7.04 (32)	7.02 (32)	7.07 (32)
Number of clinics with EHRs (% of total clinics)	16,910 (88%)	17,944 (94%)	18,386 (96%)
Number of clinics with CPOE (% of total clinics)	11,049 (58%)	12,022 (63%)	12,785 (67%)
Number of clinics in Primary Care Practices with EHRs (% of Primary Care clinics)	10,991 (88%)	11,551 (94%)	11,796 (96%)
Number of clinics in multi-hospital systems with EHRs (as % of multi-hospital systems clinics)	12,020 (90%)	13,055 (95%)	13,500 (97%)

### CPOE use

Overall CPOE use increased between 2014 and 2016, from 58 to 67%. In the vast majority of ambulatory clinics, CPOE was available both at the bedside and at the clinician station (88% in 2014 to 92% in 2016). As Table 2 illustrates, clinics that were part of a multi-hospital system (vs. those affiliated with a single hospital) had 14% (*p* < 0.0001) higher rates of CPOE use, while larger practices had an 8.7% (p < 0.0001) greater use of CPOE than smaller practices as defined by less than three physicians at the ambulatory site (see Table 2).

**Fig. 1:**
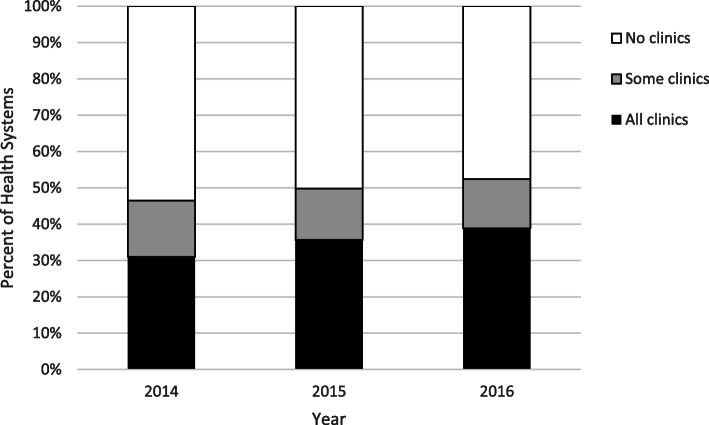
Adoption of CPOE Within Systems, 2014–2016 (4808 systems over the 3 years)

**Table 2:** Characteristics Associated with CPOE Use (Through 2016) Across All Clinics in 3 Years (54,826 observations) – Multivariable Linear Regression Model

Variable:	Primary care		Multi-hospital system		Size of practice (> 3 physicians vs. 0–3)		Year			
	Estimated coefficient	*p* value	Estimated coefficient	p value	Estimated coefficient	p value	2015	p value	2016	p value
CPOE use	0.028	0.055	**0.14**	**<.0001**	**0.087**	**<.0001**	**0.050**	**<.0001**	**0.089**	**<.0001**

**Table 3:** Characteristics Associated with CPOE Adoption During Study Period (Nonusers in 2014 Who Adopted in 2015 or 2016) Compared to Non-Adopters (7843 observations) – Multivariable Linear Regression Model

Variable:	Primary care		Multi-hospital system		Size of practice (> 3 physicians vs. 0–3)	
	Estimated coefficient	p value	Estimated coefficient	p value	Estimated coefficient	p value
CPOE adoption	0.013	0.424	**0.096**	**0.024**	**0.089**	**<.0001**

**Table 4:** Percent of Orders Placed Using CPOE, 2014–2016

	2014	2015	2016
None	10,564 (55.28%)	9549 (49.97%)	8584 (44.92%)
1–25%	113 (0.59%)	111 (0.58%)	122 (0.64%)
26–50%	304 (1.59%)	271 (1.42%)	259 (1.36%)
51–75%	1279 (6.69%)	1301 (6.81%)	1028 (5.38%)
75–94%	1856 (9.71%)	2214 (11.59%)	2982 (15.61%)
> 94%	4993 (26.13%)	5663 (29.64%)	6134 (32.10%)

Slightly more than 8000 clinics had still not yet adopted CPOE in 2014. For this sub-sample, we examined the likelihood of adoption by 2016 and found the factors that predicted adoption in 2015–2016 were being part of a multi-hospital system (9.6% increased likelihood, *p* = 0.024) and being part of a larger practice (8.9% increased likelihood, *p* < 0.0001). When looking at only those clinics with an EHR in 2014 (88%), the predictive factors were the same (data not shown). Primary care clinic type was not significantly associated with CPOE adoption (see Table 3). At the health system level, the percent of systems with *all clinics using CPOE* increased over time from 31 to 39%, and fewer health systems (from 53 to 48%) had *no adoption* among their clinics, with the *some* category remaining similar over the 3 years (see Fig. 1).

### Percent of orders placed via CPOE

Among clinics that were using CPOE, the majority (almost 60%) indicated that CPOE was used for almost all orders. The proportion of clinics using CPOE for more than 94% of their orders increased from 26% in 2014 to 32% in 2016, with a similar increase of about 6 percentage points in those using it for 75–94% of orders (see Table 4). Most of the change came from increased adoption (from clinics who were not using any CPOE at all) rather than an increase from clinics who had been using CPOE for only a small percent of orders. Very few clinics were using CPOE for less than 75% of their orders in 2014, with 8.9% doing so, a number that decreased to 7.3% in 2016. Among clinics with CPOE, in more than 90% of cases CPOE was taking place both at the point of care and at a clinician station.

### Electronic prescribing analysis

We conducted a similar analysis for medication prescribing. The two measures of interest, electronic prescribing (measured separately for both new prescriptions and refill requests) and physician order entry, were highly correlated. Using 2016 data, the variables had a Pearson correlation of 0.90 for new prescriptions and 0.88 for refills, and at least 95% of the time, a clinic had either both (indicating CPOE and e-prescribing both being used) or neither.

Analysis of e-prescribing by clinic yielded similar results. E-prescribing for new medications and e-prescribing for refill medication requests both increased over the 2-year period—from 56 to 64% for new medications and from 55 to 64% for refills. Regression analysis using linear probability demonstrated a significant relationship of e-prescribing to the kind of hospital system (those in a multi-hospital system were 16% more likely to use e-prescribing, *p* < 0.001) and larger clinic size (10% more likely, p < 0.001). As with CPOE, primary care status was not a significant predictor of use. Full results of this analysis can be found in the Additional file 1.

### Information exchange

We also found that CPOE is associated with health information exchange (HIE). Ability to exchange data across multiple vendor platforms for health information exchange (HIE) increased over the 3 years, from 31% in 2014 to 42% in 2016. Data exchange with government and hospitals also increased, from 36 to 48% and 46 to 56%, respectively—meaning that almost half of clinics still do not exchange information with hospitals for clinical information, and as of 2016, only half (51%) exchange data with other clinics for clinical information. When accounting for CPOE status, it is clear that those with electronic prescribing are also much more likely to exchange data in various ways; among the subset of our dataset that did not adopt CPOE, the rate of exchange with hospitals, for example, was 3%, compared to 80% among those with CPOE.

## Discussion

Use of CPOE among health system-affiliated clinics increased between 2014 and 2016, though near-universal adoption of CPOE, which is now the norm in the hospital setting, is far from being achieved. Although virtually all ambulatory clinics (96% by 2016) report having an EHR, only 65% reported having CPOE. Larger clinics and those affiliated with multi-hospital systems are ahead in adopting CPOE. Once adopted, CPOE quickly becomes the dominant form of ordering: clinics who have adopted it use it for more than 94% of their orders. Some health IT functionalities rely on CPOE, such as clinical decision support (CDS), so it makes sense that it would often be adopted first. Exchange of data is also associated with CPOE adoption. The benefit of CPOE on patient outcomes is likely not to be realized without other functionalities.

Despite the near-universal (96%) adoption of an EHR, about one-third of ambulatory clinics have not yet implemented one of the most basic EHR functionalities, CPOE. Adoption rates are lower in smaller practices and single-hospital systems. Therefore, policies promoting CPOE adoption should specifically target these practices. Without a bigger push, at the rate seen in these data, it will take at least another 6 years or more for CPOE to achieve the kind of use in ambulatory clinics that it already has achieved in hospitals. In particular, the “all-or-none” finding of CPOE use by clinics within system suggests that an effective action might be to encourage systems to adopt CPOE. Furthermore, the 15% or so of practices that indicated only some clinics use CPOE suggests a heterogeneity in adoption; identifying the reason for this internal variation is tolerated could help determine how to increase uptake.

Our study has limitations. The dataset includes only health system-affiliated ambulatory clinics, and as such does not allow us to estimate the prevalence of use among non-system-affiliated clinics, which likely have lower rates of adoption. Even among health system-affiliated ambulatory clinics the response rate to the survey was only 75%, meaning inference from these results to the entire sample must be made with some caution. The HIMSS data are self-reported and not validated, and there may be favorable response bias (HIMSS surveys have been shown to have higher rates of adoption in the past) [12] though they represent 75% of relevant clinics, and at the level of major functionalities, one recent validity assessment showed reasonable accuracy [13]. The HIMSS data are also restricted to ambulatory clinics affiliated with a health system; the EHR data about unaffiliated clinics has only recently been released and is still incomplete. However, other surveys do not provide the level of detail that the HIMSS survey provides [14]. Another limitation is that the response options to the survey questions about CPOE were binary—use or not—with one question on estimates of use rates (percentage of orders). The survey did not include nuances of how CPOE was implemented, what kind of tools were available, or how it was used—for example, whether it was optional or required. When the HIMSS data on unaffiliated clinics are sufficiently mature, analyses across all ambulatory clinics will be possible.

## Conclusion

CPOE use is increasing in system-affiliated ambulatory practices, but adoption is still lower than desired, given federal efforts to promote the use of EHRs and the adoption of a broad set of health IT functionalities including CPOE. When adopted, clinics tended to use it for most or all of their orders. Systems tended to have homogenous adoption or non-adoption of CPOE by the clinics within their system. As policymakers work to advance the use of health IT, a critical gap area that requires attention are smaller clinics and those affiliated with single hospital system. To determine how to support expansion efforts, it will be important to learn why these clinics are not yet using CPOE.

## Supplementary information

**Additional file 1. **Additional analysis examining two medication-related outcomes: e-prescribing for new medications and e-prescribing for refill requests. *N* = 19,804 clinics.

## Data Availability

No patient data was used in this work. The data that support the findings of this study are available from HIMSS but restrictions apply to the availability of these data, which were used under license for the current study, and so are not publicly available. Data are however available from the authors upon reasonable request and with permission of HIMSS.

## Abbreviations

CDS: Clinical decision support
CPOE: Computerized provider order entry
EHRs: Electronic health records
HIE: Health information exchange
IT: Health information technology
HITECH: Health Information Technology for Economic and Clinical Health
HIMSS: Healthcare Information and Management Systems Society
