# Supplementary material for: Trends in the use of computerized physician order entry by health-system affiliated ambulatory clinics in the United States, 2014–2016
Source: BMC Health Serv Res. 2020 Sep 7;20:836. doi: 10.1186/s12913-020-05679-4 (PMC7487802; doi:10.1186/s12913-020-05679-4)
Supplement: Supplementary file 1 — Additional file 1. Additional analysis examining two medication-related outcomes: e-prescribing for new medications and e-prescribing for refill requests. N = 19,804 clinics. [file 12913_2020_5679_MOESM1_ESM.docx]

## Appendix

Additional analysis examining two medication-related outcomes: e-prescribing for new medications and e-prescribing for refill requests. N = 19,804 clinics.

Table A1

| **Medication Management: Clinic Adoption Rates by Year** | | | |
| --- | --- | --- | --- |
|  | **2014** | **2015** | **2016** |
| Medication Management Overall | 56.87% | 61.46% | 65.30% |
| Ability to find and modify orders for all patients on a specific medication | 28.67% | 34.56% | 40.37% |
| Medication lists on-line for all patients | 45.56% | 51.21% | 55.78% |
| Medication reconciliation | 42.43% | 48.57% | 53.17% |
| e-Prescribing for new medications | 55.93% | 60.30% | 64.10% |
| e-Prescribing for refill medication requests | 54.97% | 59.69% | 63.56% |

Table A2

| **Variable:** | **Primary care** | | **Multi-hospital system** | | **Size of practice (>3 physicians vs. 0–3)** | | **Year** | | | |
| --- | --- | --- | --- | --- | --- | --- | --- | --- | --- | --- |
|  | Estimated coefficient | p value | Estimated coefficient | p value | Estimated coefficient | p value | 2015 | p value | 2016 | p value |
| e-prescribing for new medications | 0.024 | 0.084 | 0.16 | <.0001 | 0.096 | <.0001 | 0.043 | <.0001 | 0.079 | <.0001 |
| e-prescribing for refill medication requests | 0.025 | 0.082 | 0.17 | <.0001 | 0.094 | <.0001 | 0.046 | <.0001 | 0.084 | <.0001 |
